# Light Chain Cardiac Amyloidosis With Skeletal Muscle Weakness

**DOI:** 10.1016/j.jaccas.2026.108673

**Published:** 2026-09-23

**Authors:** Elyn Montgomery, Natasha Gorrie, Georgia McCaughan, James Nadel, Karan Shahi, N.K. Bart, Antonia S. Carroll

**Affiliations:** a Victor Chang Cardiac Research Institute, Darlinghurst, New South Wales, Australia; b St Vincent's Hospital Sydney, Darlinghurst, New South Wales, Australia; c Faculty of Health, University of Technology Sydney, Ultimo, New South Wales, Australia; d Faculty of Medicine and Health, School of Clinical Medicine, University of New South Wales, Kensington, New South Wales, Australia; e Garvan Institute of Medical Research, Darlinghurst, New South Wales, Australia; f Cardiology Department, St Vincent's Hospital, Darlinghurst, New South Wales, Australia; g Heart Research Institute, Newtown, New South Wales, Australia; h School of Medicine, University of Notre Dame, Sydney, New South Wales, Australia; i Faculty of Medicine and Health, Brain and Mind Centre, Sydney University, Camperdown, New South Wales, Australia

**Keywords:** cardiomyopathy, diastolic heart failure, imaging

## Abstract

**Background:**

Amyloid light chain (AL) amyloidosis can manifest with heterogeneous, multisystem features. Cardiac involvement is common and drives prognosis.

**Case Summary:**

A 54-year-old man presented with proximal muscle weakness, autonomic and gastrointestinal symptoms, and exertional intolerance. Skeletal muscle imaging suggested myositis. Cardiac evaluation revealed conduction disease, and echocardiography was consistent with infiltrative cardiomyopathy. Fluorodeoxyglucose positron emission tomography demonstrated myocardial uptake. Targeted biopsies confirmed AL amyloidosis. Plasma cell–directed therapy achieved a partial hematologic response. Given the t(11;14), the patient began a trial of a novel BCL-2 inhibitor.

**Discussion:**

Fluorodeoxyglucose-avid myocardium is described in amyloidosis but is not diagnostic. When a concurrent plasma cell clone or other red flags are present, a high index of suspicion should be maintained for AL amyloidosis.

**Take-Home Messages:**

A high index of suspicion should be maintained for cardiac AL amyloidosis in patients with atypical cardiac findings. Unexplained muscle weakness should prompt evaluation for systemic AL amyloidosis even with subtle cardiac findings.

## Visual Summary

**Figure:**
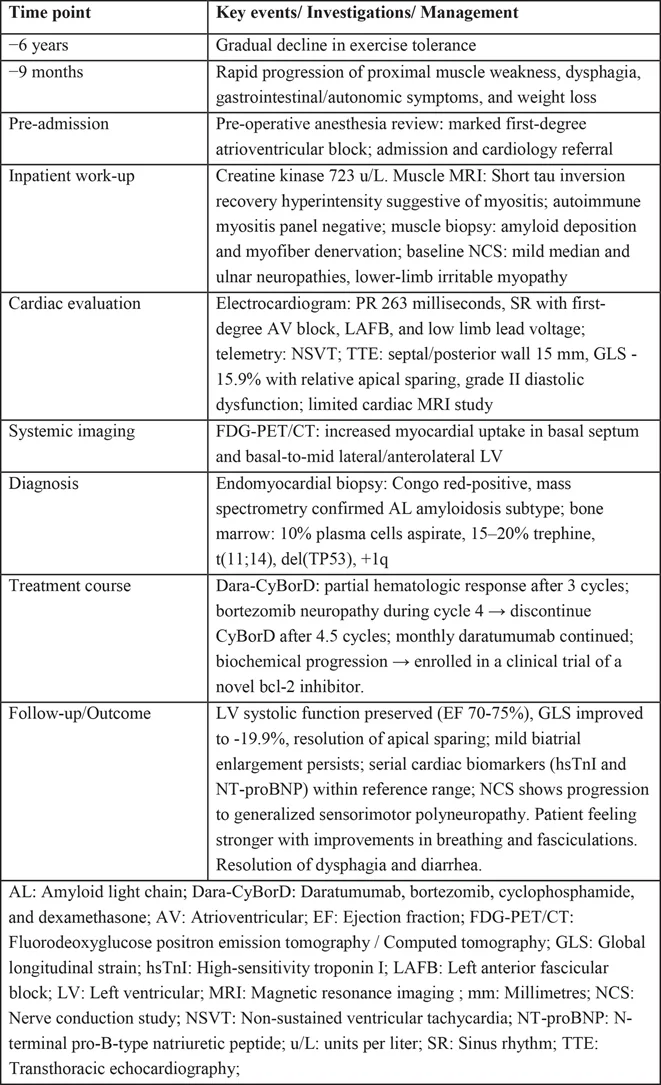

## History of Presentation

A 54-year-old man presented with rapidly progressive proximal muscle weakness, dysphagia, fasciculations, musculoskeletal pain, and exertional intolerance of 9 months duration and a 6-year history of declining exercise capacity. He described autonomic and gastrointestinal symptoms, including postural dizziness, early satiety, bloating, chronic diarrhea, 5-kg unintentional weight loss, erectile dysfunction, nocturia, and orthopnea (attributed to existing diaphragmatic dysfunction). He denied other cardiac symptoms.

Blood pressure was 136/66 mm Hg and heart rate was 87 beats/min, without postural drop. Examination demonstrated mild quadriceps wasting, hip flexion/extension rated 4/5 on the medical research council scale, reduced reflexes (1+), with preserved gait, upper limb strength, coordination, and sensation.

## Past Medical History

Past medical history included hypertension, gastroesophageal reflux disease, gout, bilateral hip arthroplasties (degenerative etiologies), hemithyroidectomy with hypothyroidism (atypical thyroid nodule), and recurrent laryngeal nerve dysfunction with right diaphragmatic dysfunction as a postoperative complication of the hemithyroidectomy.

## Differential Diagnosis

Differential diagnosis included myositis associated with connective tissue disease, malignancy, infection, or medications; sarcoidosis; amyloidosis; and myopathy (inflammatory, genetic).

## Investigations

### Neuromuscular evaluation

**Figure 1:**
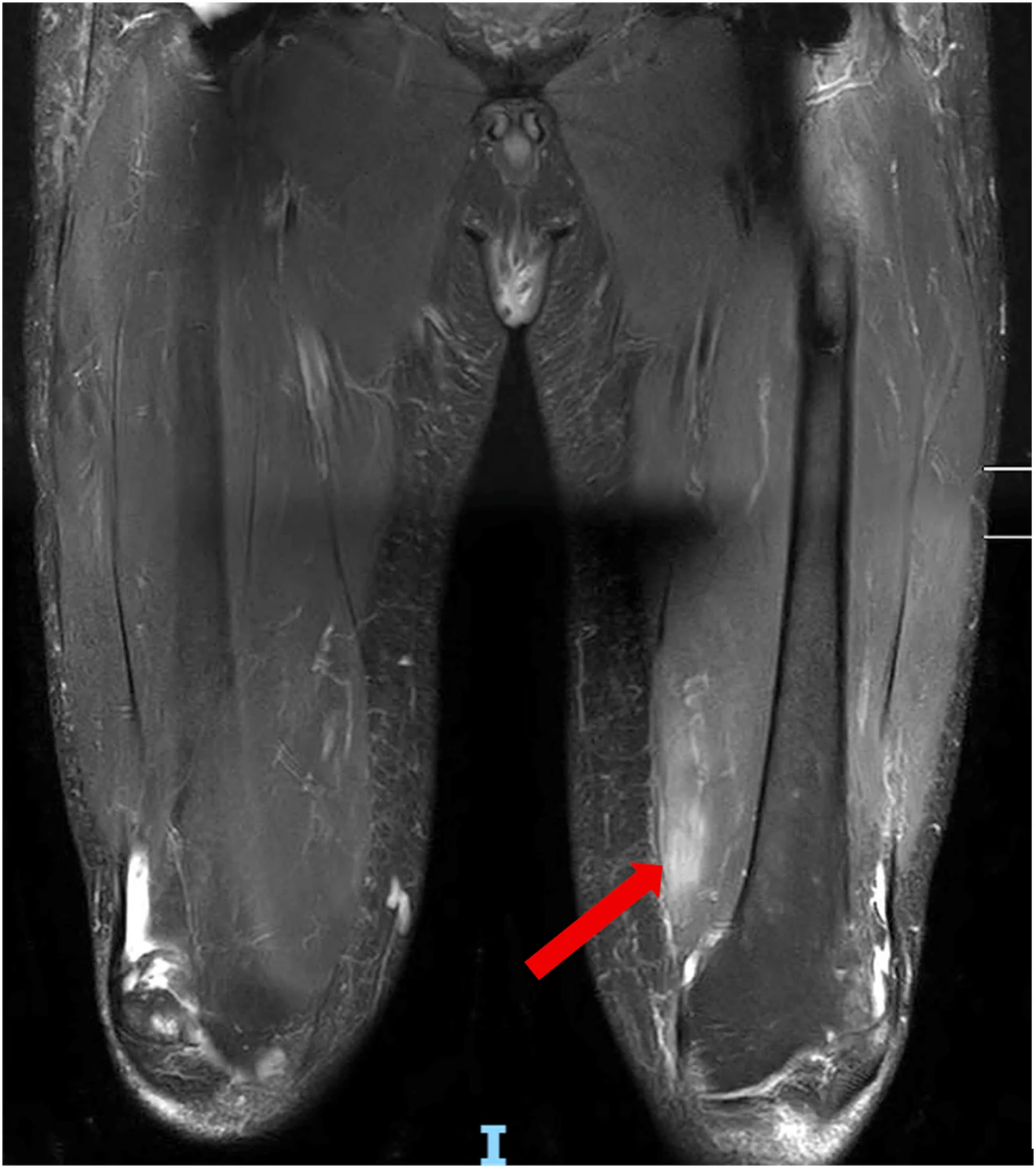
MRI of Skeletal Muscle Magnetic resonance imaging (MRI) of skeletal muscle demonstrating symmetric short tau inversion recovery hypersensitivity involving proximal lower limb musculature, consistent with an inflammatory myopathic pattern (arrow).

Baseline nerve conduction study demonstrated a mild right median neuropathy at the wrist and mild to moderate right ulnar neuropathy at the elbow, superimposed on a lower limb predominant irritable myopathy with myokymia, with no evidence of generalized large fiber polyneuropathy. Creatine kinase was 723 U/L (reference range 20-250 U/L). Magnetic resonance imaging (MRI) of skeletal muscle demonstrated symmetric short tau inversion recovery hyperintensity suggestive of myositis (Figure 1). The autoimmune myositis panel was negative. A targeted muscle biopsy revealed amyloid deposition with evidence of denervated myofibers.

### Hematologic evaluation

Initial serum free light chain (FLC) assay demonstrated an elevated lambda FLC of 583.84 mg/L, kappa FLC of 8.92 mg/L, with a difference between FLC of 574.92. Serum electrophoresis and immunofixation showed a faint band representing an immunoglobulin G lambda paraprotein. 24-hour urine protein was minimal (<0.14 g/d), but urine immunofixation detected lambda Bence-Jones (10 mg/L). Bone marrow biopsy demonstrated 15% to 20% clonal plasma cells. Fluorescence in situ hybridization revealed t(11;14), TP53 deletion, and gain of 1q.

## Cardiac evaluation

**Figure 2:**
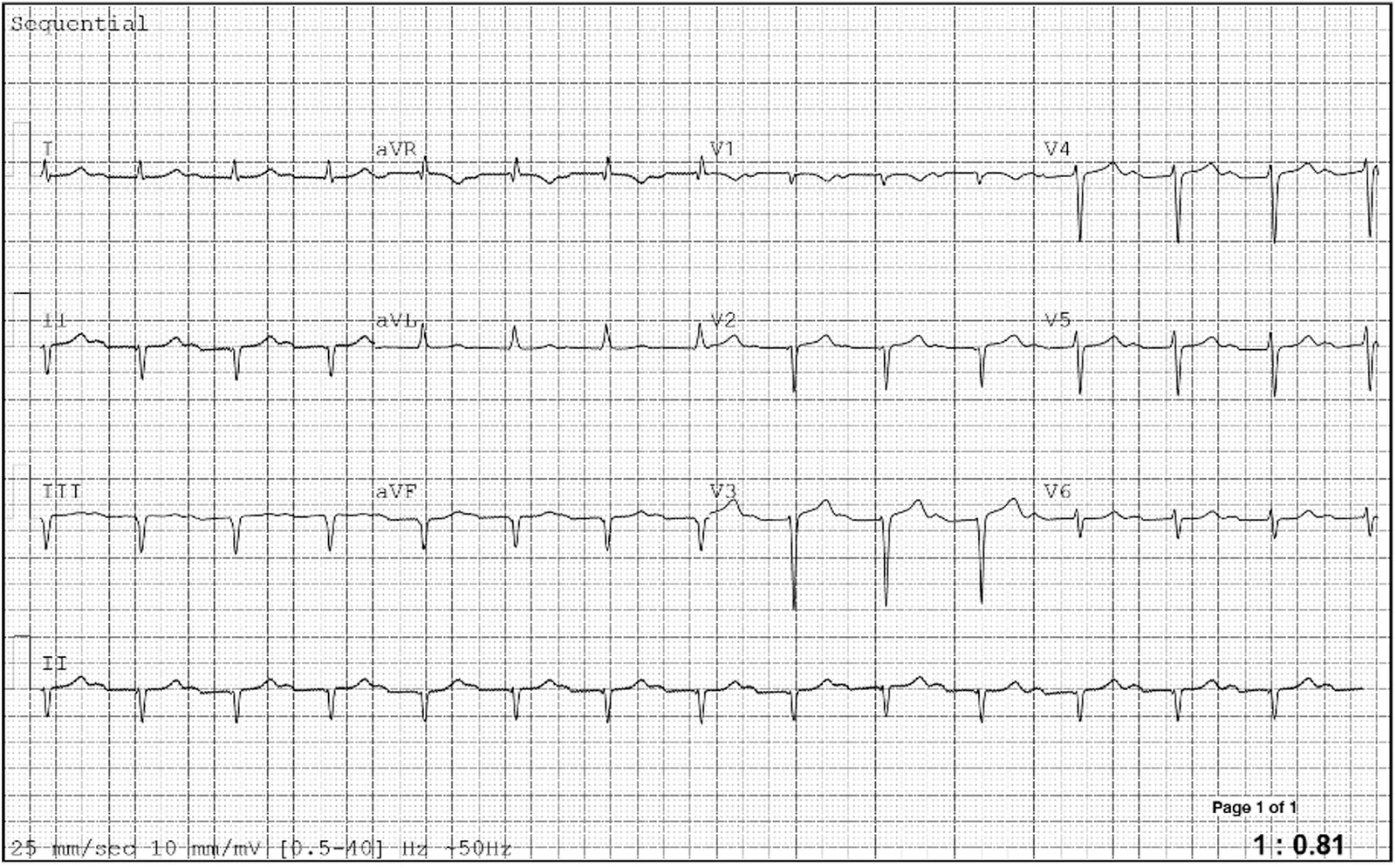
12-Lead Electrocardiogram 12-lead electrocardiogram demonstrating sinus rhythm with first-degree atrioventricular block, left anterior fascicular block, and low limb lead voltage.

**Figure 3:**
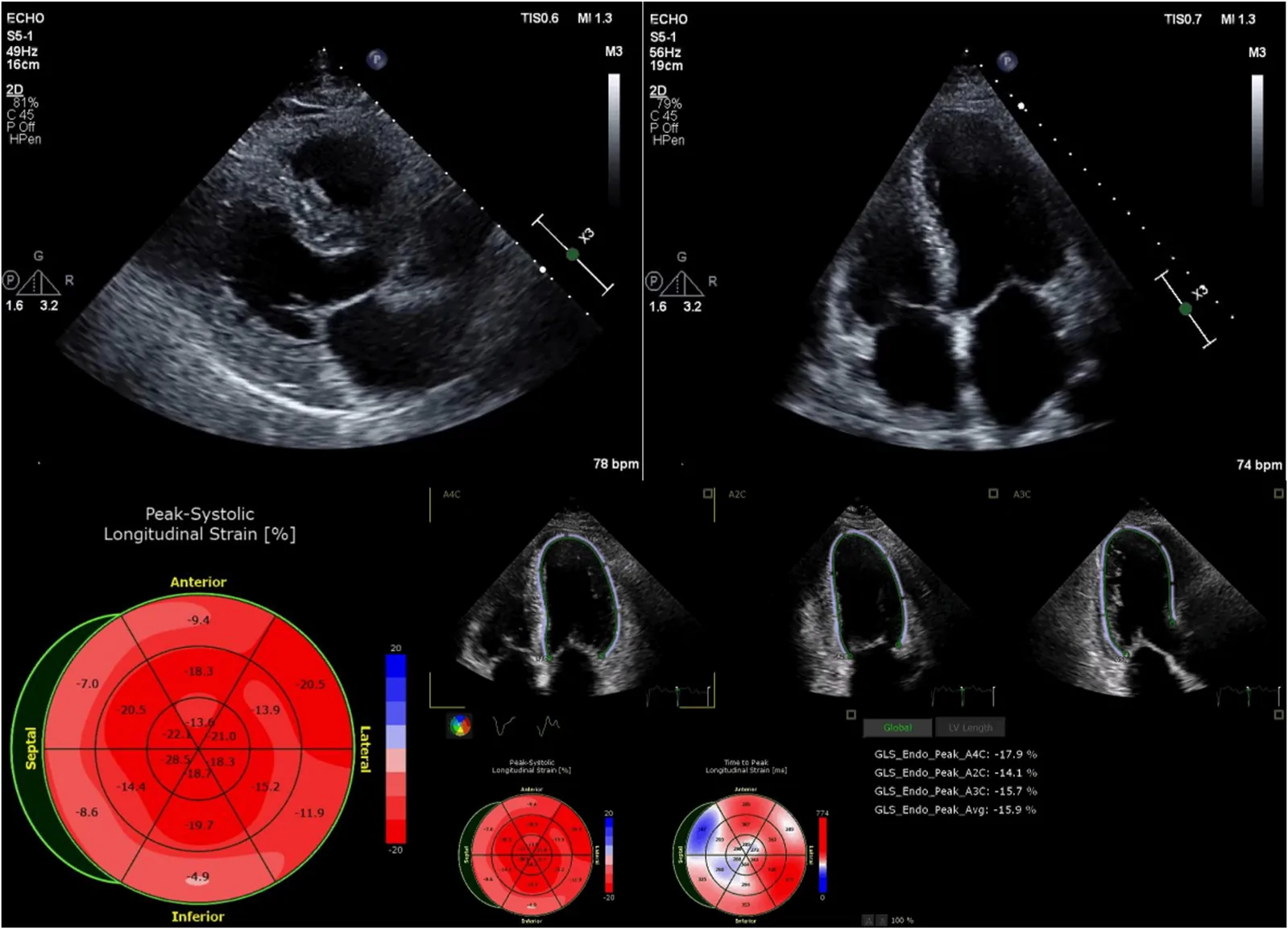
Transthoracic Echocardiogram Transthoracic echocardiogram demonstrating parasternal long axis (top left) and apical 4-chamber (top-right) views. Global longitudinal strain with reduced strain (−15.9%) and relative apical sparing (so-called cherry on top pattern) is consistent with features of cardiac amyloidosis (bottom panel).

A 12-lead electrocardiogram revealed sinus rhythm with first-degree atrioventricular block, left anterior fascicular block, and low limb lead voltage (Figure 2). Telemetry demonstrated recurrent nonsustained ventricular tachycardia. Transthoracic echocardiography showed normal left ventricular size with symmetrically increased interventricular septal and posterior wall thickness (15 mm). Left ventricular ejection fraction was normal with grade II diastolic dysfunction and reduced global longitudinal strain (−15.9%) with relative apical sparing (Figure 3).

Baseline high-sensitivity troponin I was 10 ng/L, and N-terminal pro–B-type natriuretic peptide was 294 ng/L. A cardiomyopathy gene panel did not identify any medically significant variants.

**Figure 4:**
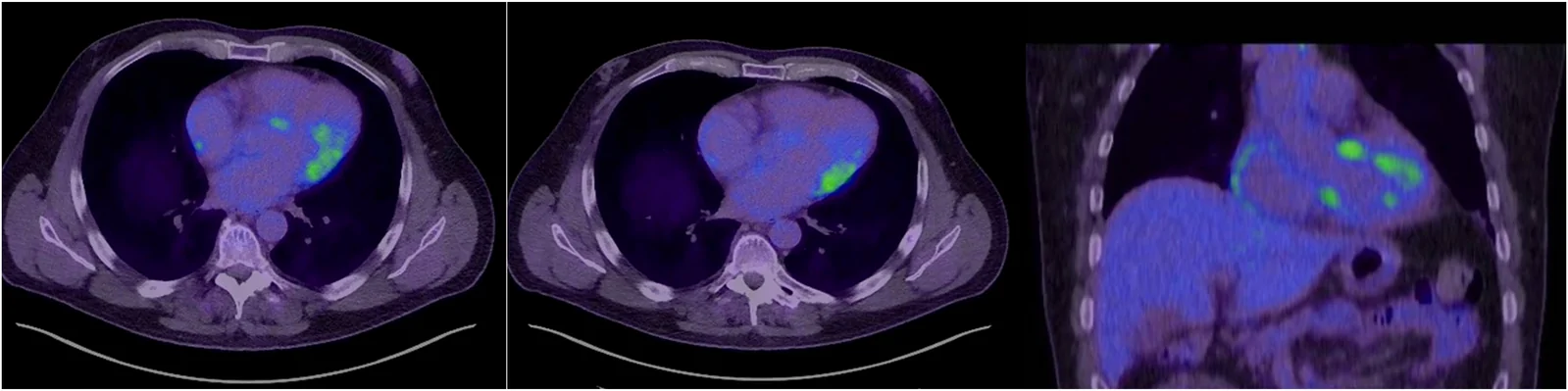
FDG-PET/CT Demonstrating Increased Myocardial Uptake Fluorodeoxyglucose positron emission tomography/computed tomography (FDG-PET/CT) demonstrates that axial cross-sectional views (left and middle panels) show uptake in the basal to mid-lateral and anterolateral segments. Sagittal view (right panel) demonstrates uptake in the basal septum.

Cardiac magnetic resonance (CMR) (limited protocol performed owing to diaphragmatic dysfunction) showed no definite late gadolinium enhancement and no significant hypertrophy. Fluorodeoxyglucose (FDG) positron emission tomography (PET)/computed tomography (CT) scan, performed to evaluate for sarcoidosis and exclude occult malignancy, demonstrated increased myocardial uptake in the basal septum and basal to mid-lateral and anterolateral segments (Figure 4).

**Figure 5:**
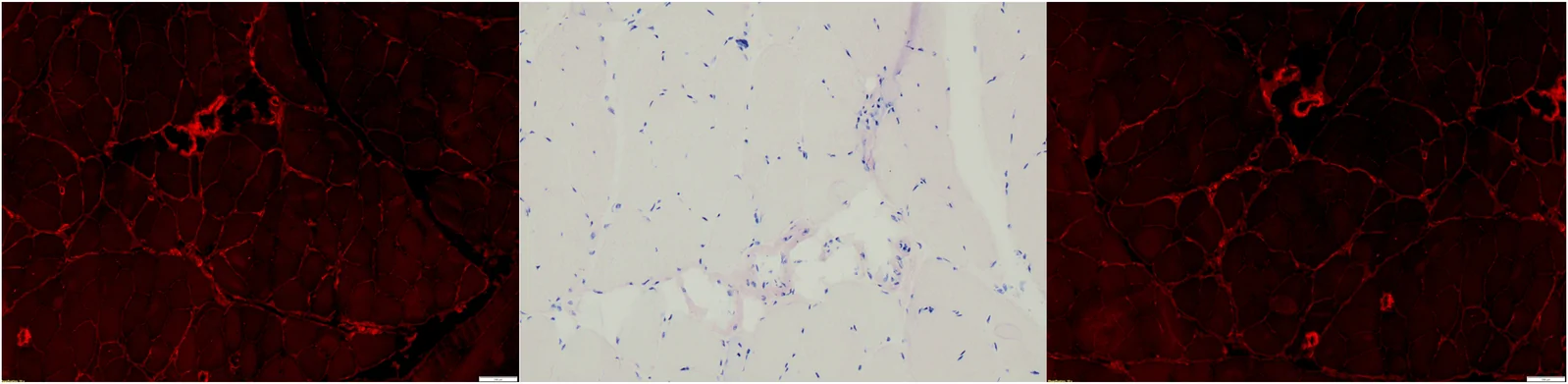
Endomyocardial Biopsy Endomyocardial biopsy specimen demonstrating amyloidosis light chain deposition disease.

Given the discordant findings for amyloid light chain (AL) amyloidosis, including nondiagnostic cardiac biomarkers and FDG-PET avidity, an endomyocardial biopsy was performed. Congo red staining demonstrated apple-green birefringence under polarized light, with deposition within myofibrils and small vessel walls (Figure 5). There was no significant inflammatory infiltration. Liquid chromatography tandem mass spectrometry detected a peptide profile consistent with AL lambda–type amyloid deposition.

Diagnostic finding was established systemic AL amyloidosis with cardiac, muscle, autonomic, and proximal nerve involvement.

## Management

Given severe conduction system disease and nonsustained ventricular tachycardia, a cardiac resynchronization therapy defibrillator was implanted. First-line plasma cell–directed therapy for AL amyloidosis was initiated with daratumumab plus bortezomib, cyclophosphamide, and dexamethasone (CyBorD).

## Outcome and Follow-Up

**Figure 6:**
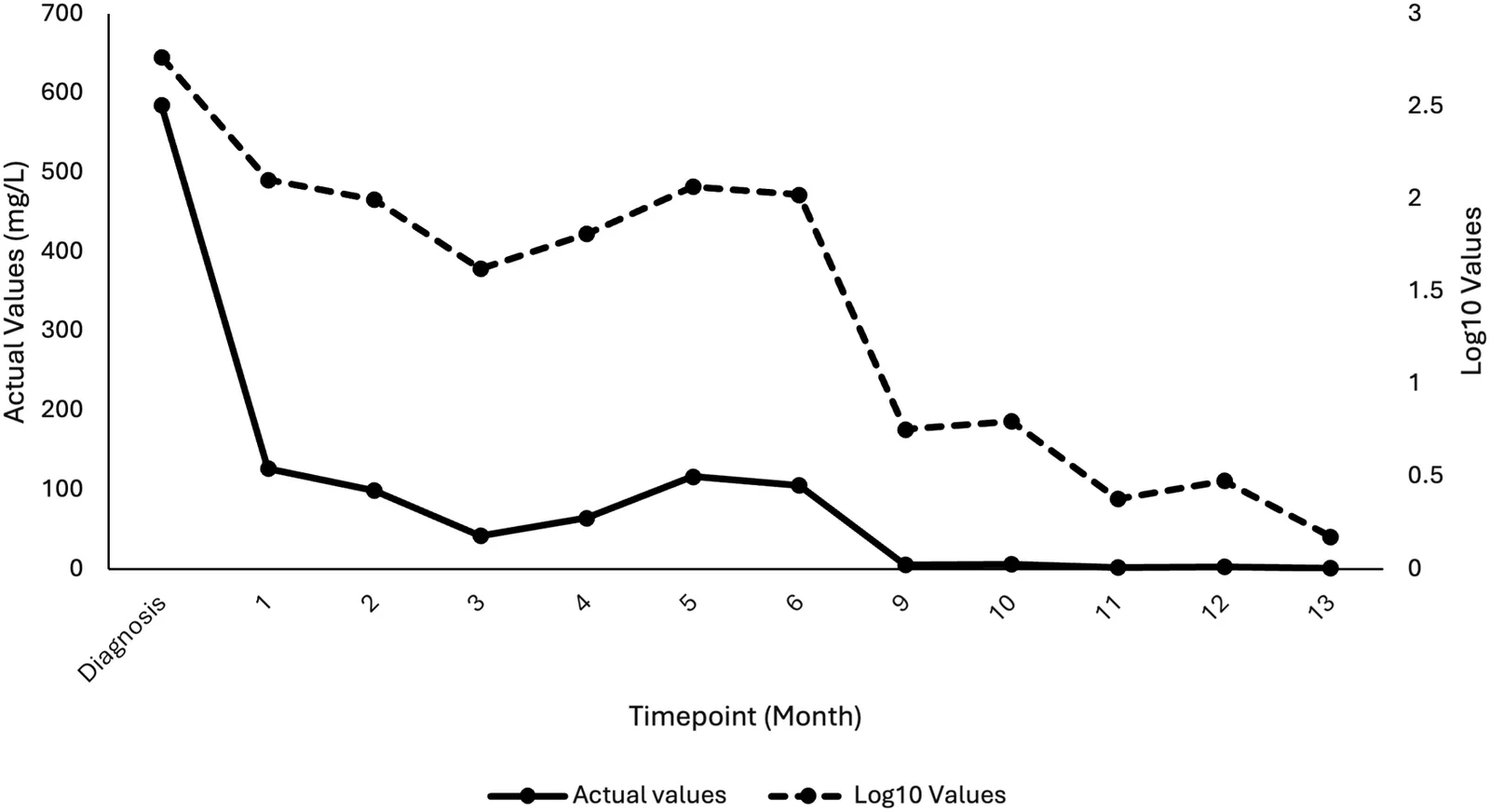
Trends in Lambda Free Light Chain Levels Trends in lambda free light chain levels from diagnosis through treatment (actual vs log10).

After 3 cycles of daratumumab plus CyBorD, the patient achieved a partial hematologic response (difference between FLC or lambda mg/L). During cycle 4, grade 1 bortezomib-induced sensory peripheral neuropathy developed primarily affecting the lower limbs. In view of the emerging neuropathy and waning response, CyBorD was discontinued. Monthly daratumumab was continued; however, lambda FLC subsequently increased on serial monitoring, consistent with biochemical progression (Figure 6). Given the presence of t(11;14), the patient was enrolled in a clinical trial of a novel BCL-2 inhibitor.

**Figure 7:**
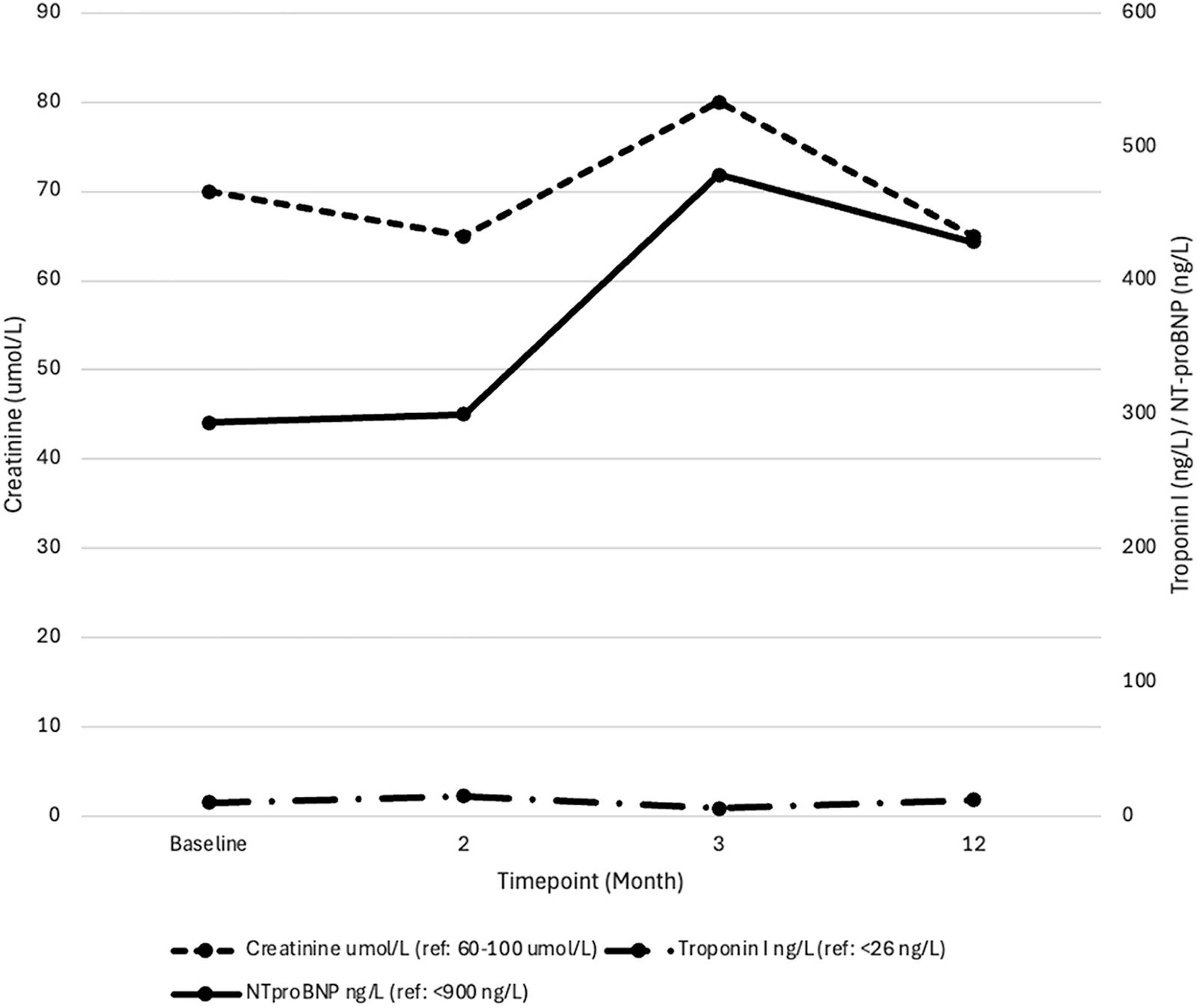
Serial Cardiac and Renal Biomarkers Serial cardiac (troponin I and N-terminal pro–B-type natriuretic peptide) and renal biomarkers (creatinine) over 12 months.

Follow-up transthoracic echocardiography showed preserved left ventricular systolic function (ejection fraction 70%) with improvement in global longitudinal strain to −19.9% and resolution of prior relative apical sparing. Increased left ventricular wall thickness persisted, with mild biatrial enlargement. Serial cardiac and renal biomarkers remained within the reference range (Figure 7).

A nerve conduction study performed 1 year following diagnosis demonstrated progression to a generalized sensorimotor polyneuropathy with predominantly axonal features, with new involvement of the left, median, ulnar, and peroneal nerves. Overall, findings were consistent with neurophysiological progression of peripheral nerve involvement, which may be secondary to both AL amyloidosis and bortezomib.

On neurological follow-up, small fiber neuropathic pain continued to limit function. However, the patient reported feeling progressively physically stronger. Following normalization of lambda FLC, the dysphagic symptoms and chronic diarrhea resolved. The patient also reported improvement in breathing and a reduction in fasciculations.

## Discussion

This case illustrates the diagnostic complexities of AL amyloidosis, an underrecognized multisystem disease with heterogeneous cardiac and extracardiac clinical manifestations. Cardiac involvement occurs in up to 70% of patients with AL amyloidosis and is the principal determinant of prognosis.^1^ However, clinical recognition is often delayed because of subtle or nonspecific findings. Current guidelines recommend prioritizing tissue biopsy over CMR when monoclonal protein studies are abnormal and cardiac biomarkers or echocardiography raise suspicion for AL cardiac involvement.^1^

In this case, skeletal myopathy preceded cardiac manifestations. Amyloid infiltration of skeletal muscle is rare but can mimic inflammatory myopathies, particularly when MRI demonstrates short tau inversion recovery hyperintensity and creatinine kinase is moderately elevated. Awareness of this pattern could be critical, as misdiagnosis may lead to unnecessary immunosuppressive therapy and delayed hematologic treatment.

Orthopnea here was attributed to diaphragmatic dysfunction rather than cardiac congestion. Although invasive assessment of left heart pressures was not performed, cardiac biomarkers were only borderline with normal diastology on transthoracic echocardiography and no clinical or imaging evidence of fluid overload. Diaphragmatic weakness, in this case, is an underrecognized noncardiac cause of orthopnea, resulting from reduced lung volumes and impaired ventilation related to supine position.

Myocardial FDG uptake created a diagnostic dilemma. FDG-PET/CT is not considered a first-line investigation for cardiac amyloidosis; however, diffuse or focal myocardial FDG uptake has been recognized as a potential imaging clue in AL cardiac amyloidosis.^2^

Case reports describe predominantly diffuse ventricular FDG uptake in biopsy-proven AL cardiac amyloidosis.^3^ The pattern can mimic inflammatory cardiomyopathies, including myocarditis or sarcoidosis, with proposed mechanisms including light chain–mediated myocyte toxicity with secondary inflammation, microvascular dysfunction, and altered myocardial metabolism, rather than true inflammation.^4^ In this case, endomyocardial biopsy confirmed AL amyloid deposition without inflammatory infiltrate, emphasizing that FDG findings should prompt targeted hematologic and histologic evaluation rather than be interpreted in isolation.

Intense biventricular FDG uptake has been described across amyloidosis subtypes, highlighting FDG-PET/CT as an incidental marker of cardiac involvement.^5^^,^^6^ Subsequent case literature has reinforced this observation, describing diffuse myocardial uptake in biopsy-proven systemic AL amyloidosis.^3^^,^^7^ However, earlier series demonstrated inconsistent myocardial FDG uptake despite frequent extracardiac involvement.^2^ This reinforces that FDG findings in suspected AL amyloidosis should be interpreted within guideline-directed diagnostic pathways.^1^

FDG-avid myocardium in AL amyloidosis is biologically plausible and increasingly reported, yet it remains nonstandard.^3^ Where FDG-PET/CT is performed, nonphysiological, diffuse myocardial uptake, should prompt a targeted AL amyloidosis work-up, including monoclonal protein testing, CMR, and histology where needed. In our case, myocardial FDG avidity helped reconcile otherwise discordant elements (limited CMR, modest biomarkers), prompting definitive endomyocardial biopsy and diagnosis.

Conduction system disease and arrhythmias were prominent in our patient despite nondiagnostic cardiac biomarkers. This discordance highlights that abnormalities in cardiac conduction and subtle functional changes may occur even in the absence of significant changes in wall thickness, strain, or N-terminal pro–B-type natriuretic peptide. Such findings may reflect predominantly small vessel cardiac amyloid causing ischemia of the conduction system or preferential amyloid deposition within the conduction system itself.^8^ This underscores the importance of integrating multidisciplinary care, multimodality imaging, protein studies, and tissue confirmation in cases with discordant findings.

This case highlights diagnostic and management challenges in atypical AL amyloidosis. Early initiation of direct plasma treatment is critical, yet treatment may be complicated by therapy-related toxicity and variable hematologic responses. Careful longitudinal follow-up, ideally coordinated through a multidisciplinary team, is essential to monitor multisystem outcomes as well as treatment-related toxicities and optimize patient care.

## Conclusions

AL amyloidosis should be suspected in patients with unexplained skeletal myopathy, autonomic dysfunction, and conduction abnormalities, even when cardiac biomarkers or imaging findings are nondiagnostic. Myocardial FDG uptake can serve as a helpful clue in this context, prompting confirmatory testing. Early recognition, timely clone-directed therapy, and coordinated multidisciplinary oversight are essential for accurate diagnosis and effective treatment, with longitudinal monitoring across the cardiac, hematologic, and neuromuscular domains vital.

## Funding Support and Author Disclosures

Dr Montgomery has received funding from St Vincent's Clinic Research Foundation, Sydney, New South Wales, Australia, and education support from Pfizer. Dr Bart has received a research grant, education support, and honoraria from Pfizer and has served on an advisory board for Pfizer, New South Wales, Australia; has received education support and honoraria from Medison Pharma and has served on an advisory board for Medison Pharma; has received honoraria from Bristol-Myers Squibb and served on advisory boards for Bristol-Myers Squibb; has served on an advisory board for Novo Nordisk; and has received a research grant from BridgeBio and has served on an advisory board for BridgeBio. Dr Carroll has received a research grant, education support, and honoraria from Pfizer and has served on an advisory board for Pfizer, New South Wales, Australia; has received education support and honoraria from Medison Pharma and has served on an advisory board for Medison Pharma; and has served on an advisory board for Alnylam. Dr Gorrie has received speaker fees, educational meeting support, and honoraria from Pfizer and has received honoraria from Novartis. Dr McCaughan has received honoraria from Johnson & Johnson Innovative Medicine and and has received honoraria from Pfizer. All other authors have reported that they have no relationships relevant to the contents of this paper to disclose.

**Take-Home Messages**

• A high index of suspicion should be maintained for cardiac AL amyloidosis in patients with atypical cardiac findings, such as modest biomarker elevation or discordant imaging.
• Unexplained skeletal muscle weakness, particularly with MRI short tau inversion recovery hyperintensity, should prompt evaluation for systemic AL amyloidosis even when cardiac findings are subtle.

**Visual Summary:** Clinical Timeline of Key Events, Investigations and Management of Light Chain Cardiac Amyloidosis

Time Point	Key Events/Investigations/Management
6 y prior	Gradual decline in exercise tolerance.
9 mo prior	Rapid progression of proximal muscle weakness, dysphagia, gastrointestinal/autonomic symptoms, and weight loss.
Pre-admission	Preoperative anesthesia review: Marked first-degree atrioventricular block; admission and cardiology referral.
Inpatient work-up	Creatine kinase 723 U/L. Muscle magnetic resonance imaging: Short tau inversion recovery hyperintensity suggestive of myositis; autoimmune myositis panel negative. Muscle biopsy: Amyloid deposition and myofiber denervation. Baseline nerve conduction study: Mild median and ulnar neuropathies, lower limb irritable myopathy.
Cardiac evaluation	Electrocardiogram: PR interval 263 ms, sinus rhythm with first-degree atrioventricular block, left anterior fascicular block, and low limb lead voltage. Telemetry: Nonsustained ventricular tachycardia. Transthoracic echocardiography: Septal/posterior wall 15 mm, global longitudinal strain −15.9% with relative apical sparing, grade II diastolic dysfunction. Limited cardiac magnetic resonance study.
Systemic imaging	Fluorodeoxyglucose positron emission tomography/computed tomography: Increased myocardial uptake in basal septum and basal to mid-lateral/anterolateral left ventricle.
Diagnosis	Endomyocardial biopsy: Congo red positive, mass spectrometry confirmed amyloid light chain amyloidosis subtype. Bone marrow: 10% plasma cells aspirate, 15%-20% trephine, t(11;14), del(TP53), +1q
Treatment course	Daratumumab plus bortezomib, cyclophosphamide, and dexamethasone: Partial hematologic response after 3 cycles; bortezomib neuropathy during cycle 4 → discontinuation of bortezomib, cyclophosphamide, and dexamethasone after 4.5 cycles; monthly daratumumab continued; biochemical progression → patient enrolled in clinical trial of a novel BCL-2 inhibitor.
Follow-up/outcome	Left ventricular systolic function preserved (ejection fraction 70%-75%), global longitudinal strain improved to −19.9%, resolution of apical sparing; mild biatrial enlargement persists; serial cardiac biomarkers (high-sensitivity troponin I and N-terminal pro–B-type natriuretic peptide) within reference range; nerve conduction study shows progression to generalized sensorimotor polyneuropathy. Patient reported feeling stronger with improvements in breathing and fasciculations. Resolution of dysphagia and diarrhea.

## References

[1] Kukreti V., Seftel M.D., Aguirre M.A. (2026). American Society of Hematology (ASH) 2026 guidelines on diagnosis of light chain amyloidosis. Blood Adv.

[2] Mekinian A., Jaccard A., Soussan M. (2012). 18F-FDG PET/CT in patients with amyloid light-chain amyloidosis: case-series and literature review. Amyloid.

[3] Hatipoglu S., Wechalekar A.D., Wechalekar K. (2023). Extensive cardiac FDG uptake in a patient with AL amyloidosis. J Nucl Cardiol.

[4] Minamimoto R. (2021). Series of myocardial FDG uptake requiring considerations of myocardial abnormalities in FDG-PET/CT. Jpn J Radiol.

[5] Gazzilli M., Bertoli M., Albano D. (2020). Cardiac amyloidosis incidentally detected by 18F-FDG PET/CT. J Nucl Cardiol.

[6] Aldajani A., Chetrit M. (2023). Diffuse 18F-FDG PET uptake in a patient with biopsy-proven ATTR cardiac amyloidosis: a potential pitfall in interpretation. CJC Open.

[7] Wang J., Doss M., Jariatu K. (2025). Increased F18-FDG uptake in the myocardium: how much is too much to be physiologic?. J Nucl Med.

[8] Hartnett J., Jaber W., Maurer M. (2021). Electrophysiological manifestations of cardiac amyloidosis: *JACC: CardioOncology* state-of-the-art review. JACC CardioOncol.

